# Exploring the Membrane Mechanism of the Bioactive Peptaibol Ampullosporin A Using Lipid Monolayers and Supported Biomimetic Membranes

**DOI:** 10.1155/2010/179641

**Published:** 2011-02-17

**Authors:** Marguerita Eid, Sonia Rippa, Sabine Castano, Bernard Desbat, Joël Chopineau, Claire Rossi, Laure Béven

**Affiliations:** ^1^UMR 6022 CNRS Génie Enzymatique et Cellulaire, Université de Technologie de Compiègne, BP 20529, 60205 Compiègne Cedex, France; ^2^CBMN, Chimie et Biologie des Membranes et des Nanoobjets CNRS, UMR 5248, Université de Bordeaux I, ENITAB, 33607 Pessac, France; ^3^CNRS, UMR 5253 Institut Charles Gerhardt, Université Montpellier 2, Ecole Nationale Supérieure de Chimie de Montpellier, Université Montpellier 1, 34093 Montpellier Cedex, France; ^4^Université de Nîmes, 30000 Nîmes, France; ^5^INRA, UMR 1090 Génomique Diversité et Pouvoir Pathogène, 33883 Villenave d'Ornon, France; ^6^Université de Bordeaux 2, UMR 1090 Génomique Diversité Pouvoir Pathogène, 33883 Villenave d'Ornon Cedex, France

## Abstract

Ampullosporin A is an antimicrobial, neuroleptic peptaibol, the behavior of which was investigated in different membrane mimetic environments made of egg yolk *L*-**α**-phosphatidylcholine. In monolayers, the peptaibol adopted a mixed **α**/3_10_-helical structure with an *in-plane* orientation. The binding step was followed by the peptide insertion into the lipid monolayer core. The relevance of the inner lipid leaflet nature was studied by comparing ampullosporin binding on a hybrid bilayer, in which this leaflet was a rigid alkane layer, and on supported fluid lipid bilayers. The membrane binding was examined by surface plasmon resonance spectroscopy and the effect on lipid dynamics was explored using fluorescence recovery after photobleaching. In the absence of voltage and at low concentration, ampullosporin A substantially adsorbed onto lipid surfaces and its interaction with biomimetic models was strongly modified depending on the inner leaflet structure. At high concentration, ampullosporin A addition led to the lipid bilayers disruption.

## 1. Introduction

Living cells naturally produce diverse membranotropic peptides displaying antifungal and/or antibacterial activities. These properties have been attributed to their molecular interaction with the target cell membranes. Three basic models have been proposed for membrane interaction mechanisms leading to membrane permeabilization. The first model consists in the formation of barrel-stave pores [1], in which the pore lumen is formed by the polar side of amphipathic and helical peptide monomers assembled into a bundle. The second major mechanism that has been proposed for membranolytic, amphipathic peptides is the formation of the so-called “toroidal” transient pores [2, 3]. In this model, the peptides bind to the polar head groups of the cell membrane phospholipids (PL) and, above a critical peptide concentration, continuously bend the lipid leaflet favouring the formation of a pore lined by both the peptides and the lipid polar headgroups. The third major model is called the “carpet-like” mechanism. While the two former modes of action require the formation of a pore structure, the latter one is subsequent to the accumulation of flat-oriented peptides at the lipid bilayer surface and may lead to membrane disruption and formation of mixed lipid-peptide aggregates [4].

The peptaibiotics' family [5, 6] comprises a large number of defense peptides, most of which share common features such as an acetylated *N*-terminal amino acid residue, a *C*-terminal amino alcohol and a high proportion of nonproteogenic residues such as *α*-aminoisobutyric acid, isovaline, hydroxyproline, or ethylnorvaline. Among peptaibiotics, peptaibols are rich in *α*-aminoisobutyric acid and carry a *C*-terminal residue corresponding to a 1,2-aminoalcohol [7–10]. Information about the natural sources and the structural properties of more than 300 peptaibiotics is available in the peptaibol databank [11]. Most peptaibols are bioactive and interact with cell membranes. Ampullosporin A (AmpA) is a 14-amino acid residue peptaibol that exhibits a wide spectrum of biological activities. On the one hand, its antimicrobial effects [12] and its capacities to induce the volatile compounds emission in Lima bean plants [13], to elicit at high concentration the rRNA-cleavage-associated death [14] and at lower dose a hypersensitive-like response in *Arabidopsis* plantlets [15] are shared with longer natural peptaibiotics such as alamethicin (Alm). On the other hand, induction of pigment formation in *Phoma destructiva* and neuroleptic activities in mice [12, 16] have been demonstrated for the “medium-sized” peptaibols AmpA and zervamicin IIA-B (15 amino acid residues) [17], but not for the longer ones. 

The exact molecular mechanism responsible for peptaibols' biological activities is not fully understood. Nevertheless, membrane biomimetic systems have been widely used in order to decipher the mechanism responsible for the insertion of the long peptaibol Alm in membranes. This peptaibol has been extensively studied, using various biophysical methods such as oriented circular dichroism, solid-state NMR, fluorescence, Raman spectroscopy, X-Ray diffraction, differential scanning calorimetry, fluorescence recovery after photobleaching (FRAP), electron paramagnetic resonance [18–25]. It is now established that Alm forms barrel-stave transmembrane pores [26]. The mechanism by which Alm incorporates into membranes is highly cooperative [27] and shows dependency upon voltage with a poor cation selectivity in both artificial [28] and natural membranes [29]. To date, among the naturally occurring antimicrobial peptides, only Alm mechanism proved to conform to the barrel-stave model [3].

The mechanism of AmpA with sequence Ac-WAUULUQUUUQLUQ-Lol (where Ac: acetyl, U: *α*-amino-isobutyric acid and Lol: Leucinol) is less clear. As for Alm, a correlation between AmpA bioactivities and its ability to permeabilize membranes has been suggested [30, 31]. AmpA interacts with egg yolk *L*-*α*-phosphatidylcholine (eggPC) vesicles in the absence of membrane voltage [32]. Its length (23 Å in the crystals) [33] has to be compared to the hydrophobic core of natural membranes typically having a thickness ranging from 30 to 40 Å [34]. AmpA forms voltage-dependent ion conducting pores in eggPC planar bilayers [31], the hydrophobic core thickness of which has been estimated to be 26 Å by others [35]. Nevertheless, the conductance changes monitored in the presence of AmpA, in comparison with those observed in the presence of Alm [31], are of weak amplitude. As recently suggested [17], the specific activities of the medium-sized peptaibols zervamycin and AmpA may be based on similar mechanisms. Their voltage-dependent pore formation abilities being insufficient to explain their specific activities, an additional, voltage-independent membrane mechanism might exist.

Although the studies previously undertaken on various artificial lipid platforms and using various analytical techniques [32, 36] brought invaluable information on the mode of interaction of AmpA with membranes, the coupling of analytical techniques such as FRAP and SPR on complementary membrane models and under comparable experimental conditions (i.e., type and concentration of buffer system) can prove very useful to examine the various steps of the peptaibol membrane mechanism. Among the available membrane models, supported lipid membranes [37, 38] allow studying peptide interaction at the lipid/water interface. Examples of complementary supported membrane models that constitute powerful tools to quantitatively characterize the binding of extrinsic and integral membrane proteins to membrane lipids are the hybrid bilayer membranes (HBM) and the tethered-bilayer-lipid-membranes (*t*-BLM) [39]. HBM has successfully been used to examine the membrane mechanism of membranotropic, cationic peptides [40]. The potentialities of the *t*-BLM compared to the other existing supported designs have been demonstrated for membrane proteins [41], but this model remains to be evaluated for peptide-membrane interaction studies. 

In this work, with the aim of studying AmpA membrane behaviour in the absence of voltage, investigations of AmpA molecules orientation and conformation in lipid environments were first carried out with eggPC monolayers at the air/water interface by using Brewster angle microscopy (BAM) and polarization modulation infrared reflection absorption spectroscopy (PMIRRAS) as analytical tools. Supported EggPC planar mono- and bilayers were then made profitable to investigate AmpA effect on lipid mono- and bi-layers' fluidity as a function of total peptide concentration using FRAP and to examine AmpA membrane binding properties by SPR studies. To the best of our knowledge, this is the first report of membrane binding and insertion studies of a hydrophobic, membranotropic peptide combining SPR and FRAP analyses on both models HBM and *t*-BLM.

## 2. Materials and Methods

### 2.1. Materials

Ampullosporin A (AmpA) isolated from **Sepedonium ampullosporum** (HPLC purified to ≥98%) was purchased from Alexis (Lausanne, Switzerland). Stock solutions of AmpA (10 mM) in methanol were kept at −20°C and diluted in buffer solution just before use. EggPC type XVI-E, 1,2-dipalmitoyl-sn-glycero-3-phosphoethanolamine-N-7-nitro-2,1,3-benzoxadiazol-4-yl (DPPE-NBD used as fluorescent probe), N-octyl-*β*-D-glucopyranoside, bovine serum albumin and 2-mercaptoethylamine (cysteamine hydrochloride, ≥99%) were purchased from Sigma-Aldrich (St Quentin-Fallavier, France). 1,2-distearoyl-sn-glycero-3-phosphoethanolamine-poly-(ethyleneglycol)-N-hydroxysuccinimide (DSPE-PEG_77_-NHS, 77 ethylene glycol units) was from Shearwater Polymers (Huntsville, AL, US) and aminopropyl-dimethylethoxysilane (99%) from ABCR (Karlsruhe, Germany). Glass microscope slides were from Menzel-Glaser (Braunschweig, Germany). All other chemicals used in this work were of analytical grade. All buffers prepared from Milli-Q water (resistivity higher than 18.2 MΩ·cm^−1^) were filtered and thoroughly degassed. The standard buffer (HEPES-NaCl buffer) was 20 mM N-[2-hydroxyethyl] piperazine-N′-[2-ethanesulfonic acid] (HEPES), pH 7.5, 150 mM NaCl. 

### 2.2. Film Formation for Surface Pressure Measurements

Monolayer studies were carried out on a computer controlled Langmuir film balance (Nima Technology, Coventry, England) and the surface pressure was measured by the Wilhelmy method [42] using a filter paper plate. The trough (*V* = 60 mL, *S* = 105 cm^2^) made of Teflon was filled with the buffer made using ultrapure water. The experiments were performed at 25°C. The monolayer was formed as previously described [43] by spreading first 20 *μ*L of a 1 mg·mL^−1^ eggPC chloroform solution at the air/water interface. After complete evaporation of the solvent (*≈*15 min), the monolayer was slowly and continuously compressed up to 30 mN·m^−1^. Mixed peptide/lipid films were obtained by injection of few *μ*L of methanolic AmpA stock solution in the buffered subphase that define the total AmpA concentration.

### 2.3. BAM Measurements

The interaction of AmpA with the lipid monolayer at the air/water interface was observed using a Brewster angle microscope (NFT BAM2plus, Göttingen, Germany) mounted on the Teflon Langmuir trough and equipped with a frequency doubled Nd:Yag laser (532 nm, 20 mW), polarizer, analyzer, and a CCD camera. The spatial lateral resolution of the BAM was 2 *μ*m and the size image 600 ∗ 450 *μ*m with the ×10 lens. The exposure time was adjusted to avoid the camera saturation. The BAM software included in the BAM instrument was used for the determination of the layer thickness. The BAM measurements calibration was made using the linear function that exists between the reflectance and the gray level (GL). To establish this function, the experimental curve of the GL as a function of the incidence angle was compared to the Fresnel curve that can be fitted by a parabola around the Brewster angle minimum. The reflectance value, the experimental Brewster angle, and the optical index of the film were inserted in the BAM thickness model to evaluate the thickness of the layer at the surface [44].

### 2.4. PMIRRAS Spectroscopy

PMIRRAS spectra were recorded on a Nexus 870 spectrophotometer equipped with a photovoltaic HgCdTe detector (SAT, Poitiers, France) cooled at 77 K. 200 or 300 Scans were recorded at a resolution of 4 or 8 cm^−1^ after injection of different concentrations of AmpA into the buffered subphase. In PMIRRAS experiments, the IR beam is quickly modulated between the *p* and *s* polarization, and the sum and difference interferograms are processed and Fourier-transformed to yield the differential reflectivity:

(1) $$\frac{\Delta R}{R}=\frac{\left(R_{p}-R_{s}\right)}{\left(R_{p}+R_{s}\right)J_{2}}.$$

The liquid water absorption contribution and the dependence on Bessel functions *J*_2_, were removed by the spectra division by those of the subphase. The external beam was focused on the sample with a mirror at an optimal incident angle of 75°. In this case, transition moments oriented in the plane of the interface give intense and upward oriented bands [45].

### 2.5. Substrate Surface Preparation

All glass surfaces were carefully cleaned in a sulfochromic acid solution (5% v/v potassium dichromate in sulfuric acid, caution: this solution reacts violently with organics and should be handled with extreme care), thoroughly rinsed and stored in Milli-Q water. Before use, the glass surfaces were dried at 110°C for 20 min. *Self-assembled monolayers on gold (SPR measurements):* the gold surfaces were prepared from deposition of a gold layer of 47 ± 1 nm on glass slides by thermal evaporation under vacuum (Evaporator Edwards Auto 306, rate 0.01 nm·s^−1^, pressure 2 × 10^*‒*0^ mbar). For hybrid bilayer construction, a 1-octadecane thiol monolayer was self-assembled on top of the gold surface. This coating was obtained by adsorption of 1-octadecane thiol (1 mM in ethanol, water (4/1) solution) onto bare gold. The coated slides were thoroughly rinsed with toluene, ethanol, and MilliQ water and dried under a nitrogen stream. For the construction of the tethered bilayer, the gold substrates were functionalized by overnight immersion in a 2-mercaptoethylamine degassed solution (5 mM in pure ethanol). The coated slides were thoroughly rinsed with ethanol and dried under a nitrogen stream. *Self-assembled monolayers on glass (FRAP measurements): * for hybrid bilayer construction, a monolayer of 1-octadecyltrichlorosilane (OTS) was obtained by alkylation in a freshly prepared OTS solution (2% (w/w) in anhydrous hexadecane for 15 min. The coated glass slides were extensively rinsed with toluene and ethanol and finally dried under a nitrogen flow. For tethered bilayer construction, the glass slides were silanized by immersion into an aminopropyl-dimethylethoxysilane solution (2%, v/v) in toluene for 4 h, thoroughly rinsed with toluene, chloroform, ethanol, and water before being dried in an oven at 110°C.

### 2.6. Supported Lipid Membrane Formation

In this work, the three membrane models HBM, supported membrane bilayer (SLB), and *t*-BLM were used. Vesicles were obtained from a dried lipid film formed by removing the solvent from an eggPC chloroform solution under a nitrogen stream followed by two hours drying under vacuum. The dried lipid film was suspended in buffer. *Tethered bilayer formation (*t*-BLM):* DSPE-PEG_3400_-NHS was incorporated at 5% (w/w) in the initial chloroform solution. The lipid suspension in buffer was extruded 19 times through 50 nm size calibrated polycarbonate membranes using a syringe-type extruder (Liposofast, Avestin Inc., Ottawa, Canada). 390 *μ*L·cm^−2^ of eggPC/DSPE-PEG_3400_-NHS vesicles (1 mg·mL^−1^) in buffer were injected into the flow cell used for further SPR measurements. After 2 hours of vesicles contact with the activated surface, the cell was rinsed overnight with the buffer at a flow rate of 0.5 mL·min^−1^. *Hybrid bilayer (HBM): *the vesicles were obtained from ultrasonic irradiation (4 cycles of 3 min at 100 W each separated by a 3 min period). The HBM formation was performed by deposition of 390 *μ*L·cm^−2^ of an eggPC vesicle suspension (1 mg·mL^−1^) onto the alkyl monolayer. After 1 hour, the cell was flushed with the buffer at a flow rate of 0.5 mL·min^−1^. *Supported lipid bilayer *(*SLB*): vesicles were obtained using ultrasonic irradiation (4 cycles of 3 min at 100 W each separated by a 3 min period). The SLB formation was performed by deposition of 390 *μ*L·cm^−2^ of an eggPC vesicle suspension (1 mg·mL^−1^) onto an ultracleaned glass surface. After 1 hour, the cell was flushed with the buffer at a flow rate of 0.5 mL·min^−1^.

### 2.7. SPR Measurements

A homemade set-up (He-Ne laser beam (*λ* = 633 nm, 10 mW)) in the Kretschmann configuration was used [46]. The measurements were performed in the configuration described in the previous studies [47, 48]. Optical thicknesses were determined according to Fresnel equations using the Winspall program (Max-Planck Institute for Polymer Research, Mainz, Germany). We used the value of *n* = 1.5 for PL layers [49, 50]. To determine the amount of bound peptides, optical thicknesses were given assuming a refractive index value of *n* = 1.5 for the peptide layer [51]. AmpA solution (0.5-1-2-5-8-10 *μ*M) were injected sequentially in the cell measurements. After 60 min, the cell was flushed during 15 min with the buffer at a flow rate of 0.5 mL·min^−1^. Kinetics were measured at a fixed angle of 1° below the minimum angle. All measurements were performed at 25°C. 

### 2.8. FRAP Analyses

The diffusion coefficient of lipids was determined using FRAP. A confocal scanning light microscope (LSM 410, Micro Systems Zeiss, Germany) was used. For fluorescence measurements, an NBD fluorescent probe (DPPE-NBD) was added into the vesicles at a 2% molar ratio. AmpA solutions were injected on the top of the membrane models, with increasing concentrations from 0.5 to 10 *μ*M while respecting the identical contact time of peptide solution/lipid membrane as for SPR measurements. A buffer rinsing step was performed in between each injection. The lipid diffusion coefficients were determined from FRAP experiments according to calculations previously published [47, 48].

## 3. Results

### 3.1. Monolayer Experiments

Langmuir monolayers deposited at the air/water interface were used to detect the first steps of AmpA binding to membranes. Indeed, these simplified membrane models mimic a single lipid leaflet and allow the discrimination between the first binding steps of membranoactive peptides from the subsequent structural membrane changes [52]. This section deals with thermodynamic measurements, microscopy analysis (BAM) and PMIRRAS studies. 

**Figure 1:**
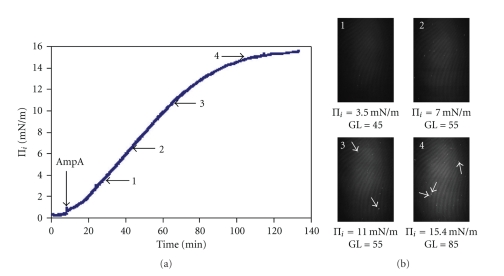
Adsorption of AmpA at the air/water interface. (a) Adsorption isotherm (surface pressure Π_*i*_ versus time) of AmpA at the air/water interface. AmpA was injected at a total concentration in the subphase of 250 nM. (b) Brewster angle microscopy images at the air/water interface recorded at the timepoints indicated by the numbered arrows in (a). GL: gray levels obtained with a shutter speed of 50 s^−1^. Arrows on photographs point to the peptide aggregates.

**Figure 2:**
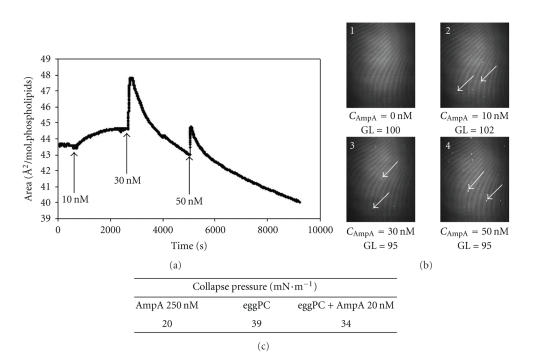
Interaction of AmpA with eggPC monolayers at the air/water interface. (a) Kinetic (area versus time) analysis of AmpA insertion in eggPC monolayers at the air/water interface for different peptide subphase concentrations at *P* = 30 mN·m^−1^. Arrows indicate AmpA injection times. (b) Brewster angle microscopy images at the air/water interface recorded after injection of AmpA at increasing concentrations. GL: gray levels obtained with a shutter speed of 50 s^−1^. Arrows on photographs point to the mixed lipid-peptide aggregates. (c) Collapse pressure values were measured upon compression of the film formed at the air/water interface by monitoring pressure-area isotherms.

In the course of this study, the surface pressure at the air/water interface was measured when pure AmpA was step wisely injected in the HEPES buffered phase. AmpA promoted a gradual increase in the surface pressure at constant area (Figure 1(a)). The higher the peptide concentration in the buffered subphase, the faster its adsorption at the interface and the higher the maximum surface pressure attained. For the highest tested peptide concentration (0.25 *μ*M), the equilibrium surface pressure reached 15.5 mN·m^−1^ on HEPES-NaCl subphase. To better understand the topography of the pure peptide monolayer, BAM images and the corresponding GL were monitored (Figure 1(b)). The BAM images showed the occurrence of few bright spots and a significant increase in the GL of the entire field upon AmpA addition. An averaged monolayer thickness of 12.2 Å could be estimated using the BAM software and a refractive index of 1.50 for AmpA. When the pure peptide monolayer was submitted to compression, the Π*-A* isotherm indicated that the maximal pressure (i.e., collapse pressure) that could be reached was around 20 mN·m^−1^(Figure 2(c)). Applying a higher pressure triggered a drastic decrease in the molecular area. This event was correlated with a reduction of the BAM images GL (data not shown).

Pure lipid monolayers at the air/water interface were obtained by deposition of eggPC in the HEPES buffered phase and relaxation of molecules was allowed to occur. A surface pressure Π = 30 mN·m^−1^ was applied to mimic the lateral pressure of natural membranes, and an initial molecular area of 43.7 Å^2^ was obtained (Figure 2(a)). BAM images of the pure lipid monolayer were homogeneous (Figure 2(b), picture 1) and an averaged thickness of 23 Å was estimated at *P* = 30 mN·m^−1^, coherent with the eggPC acyl chains length. The Π*-A* isotherm indicated a collapse pressure around 39 mN·m^−1^ (Figure 2(c)). Upon higher compression, tridimensionnal lipid reorganization was observed. AmpA injected at 10 nM in the aqueous subphase under an eggPC monolayer compressed and stabilized at 30 mN·m^−1^ promoted an increase in the molecular area (Figure 2(a)). For 30 nM AmpA, the area first reached a value of 4.3 A^2^/molecule, while the averaged normalized GL values were only slightly enhanced. The BAM images revealed a surface topography change with the occurrence of small domains, the number of which increased with AmpA concentration and which showed a distinct GL as compared to the rest of the monolayer (Figure 2(b)). Within the few seconds following the injection, 30 nM or 50 nM AmpA triggered a significant reduction of the molecular area (Figure 2(a)) below the basal level. Addition of up to 20 nM AmpA led to a significant increase in the molecular area without the following decreasing step (data not shown). Consequently, the Π*-A* isotherm of the mixed PL/AmpA film was then followed for this concentration. After addition of 20 nM AmpA, the monolayer showed a collapse pressure of 34 mN·m^−1^ (Figure 2(c)).

**Figure 3:**
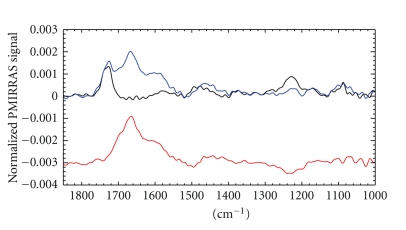
PMIRRAS spectra of the peptide-free eggPC monolayer at the air/water interface (in black) and of the AmpA-containing eggPC monolayer (in blue). AmpA concentration was 20 nM. The red curve corresponds to the difference spectrum corrected by the dilution effect.

PMIRRAS studies were carried out to determine AmpA conformation and orientation in the eggPC film at the air/water interface (Figure 3). The eggPC spectra showed the characteristic bands around 1730, 1462, 1225, and 1080 cm^−1^, respectively, due to *ν*(C=O) ester, *δ*(CH_2_) of the acyl chains and *ν*_as_ ( PO_2_^*‒*^) and *ν*_s_ (PO_2_^*‒*^) of the phosphate groups [53]. Changes induced by AmpA could be visualized on the difference spectrum (Figure 3, red curve) between the eggPC monolayer with (Figure 3, blue curve) and without (Figure 3, black curve) AmpA. A very intense amide I band was observed at a high frequency of about 1665 cm^−1^. Conversely, the amide II band (1525 cm^−1^) was reduced, resulting in a high intensity ratio of the amide I/amide II bands that was coherent with a predominant *α*-helical conformation for AmpA. A detailed examination of the amide I bandshapes indicated the occurrence of a small positive shoulder at 1670 cm^−1^ [54] suggesting a partial 3_10_-helix conformation [55] of AmpA. A second shoulder at 1595 cm^−1^ was attributed to a change in water dispersion that occurred upon AmpA interaction with the lipids. In addition, the substracted spectrum corrected by the dilution effect (Figure 3, red curve) displayed a negative band around 1225 cm^−1^, corresponding to the decrease of the lipid *ν*_as_ (PO_2_^*‒*^) intensity and thus corresponding to the perturbation of lipid organization upon AmpA interaction. Since an eggPC film is highly fluid, no significant change was observed on the v(CH_2_) in the 2800–3000 cm^−1^ region upon AmpA interaction.

### 3.2. Supported Lipid Mono- and Bilayers Experiments

#### 3.2.1. AmpA Binding

**Figure 4:**
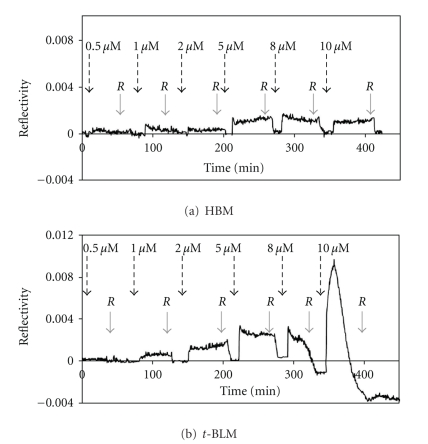
Kinetic analysis of AmpA interaction with membranes using SPR. (a) Kinetics (reflectivity versus time) of AmpA interaction were measured with the hybrid bilayer, and (b) with the tethered lipid bilayer. The signal related to the mono- or bilayers was used as the baseline. Dashed arrows indicate the peptide injection and solid arrows the buffer rinsing (*R*) steps.

The AmpA binding behaviour was investigated by SPR using two biomimetic membrane constructs. The artificial membranes formation was directly performed in the SPR cell. HBM was constituted by a continuous and fluid lipid monolayer formed on the top of a dense, rigid, and hydrophobic self-assembled submonolayer [56]. The second model was based on a tethered lipid bilayer architecture, in which the bilayer is decoupled and anchored to an amine functionalized surface by poly(ethylene)glycol (PEG) chains. The formation process and the properties of these two membranes are well known and characterized [41, 57, 58]. In the HBM model, the 1-octadecane thiol “inner” monolayer thickness was 29 ± 2.5 Å. Upon eggPC vesicle injection, the spontaneous fusion of the lipids with the support surface led to an additional thickness of 22 ± 2.5 Å and a HBM overall thickness of 50 ± 5 Å. The PEG-tethered bilayer was constructed by deposition on an amine coated surface of eggPC vesicles containing DSPE-PEG_77_-NHS lipids. The overall thickness of the *t*-BLM bilayer was 58 ± 2 Å. The AmpA solution was injected in the SPR cell for 50 min of interaction at increasing concentrations ([AmpA] = 0.5, 1, 2, 5, 8 and 10 *μ*M). The reflectivity variations observed after the successive injections of AmpA are shown in Figure 4. At the end of each injection, the bilayer was rinsed with buffer. Reproducible kinetics were obtained, but the sensorgrams could not accurately be fitted using a 1 : 1 Langmuir or a two-step binding thickness model and no relevant affinity constants could be determined. However, the thickness changes during the binding of AmpA onto the membrane could be evaluated. Due to the lipid desorbing from the support upon rinsing, values of the optical thicknesses had to be considered both before and after buffer rinsing. Data corresponding to 50 minutes peptide injection are summarized in Table 1. Before buffer rinsing, AmpA was found to adsorb on both biomimetic constructs. For concentrations of AmpA below 5 *μ*M, the maximum binding response increased as a function of peptide concentration. AmpA binding led to higher association levels with the *t*-BLM compared to the HBM, by around a two-fold magnitude order (Table 1). After buffer flushing, the resonance signal returned to its initial value that corresponded to the initial thickness level for both bilayers. Flushing very probably triggered the leakage from the support of most of the bound peptides. Nevertheless, the refractive index used for optical thickness calculations being the same for lipids and peptides, remaining, deeply membrane-inserted peptides may not be excluded after flushing. For concentrations of AmpA higher than 5 *μ*M, the binding events strongly depended on the membrane model. The signal obtained with the HBM reached a maximum level that corresponded to an additional layer thickness of 2.5 Å. After buffer rinsing and peptide desorption, the SPR signal returned to the baseline level. Conversely, using the *t*-BLM, injection of AmpA at high concentrations led to a biphasic reflectivity curve. The first phase corresponded to a fast and important increase of the SPR response. In the second part, the signal rapidly decreased and dropped beyond the baseline level, indicating a lipid departure from the support. The thickness values could then only be estimated after buffer rinsing to ensure enough signal stability. For 8 *μ*M and 10 *μ*M AmpA, the lipid loss led to a reduction of the averaged optical bilayer thickness of 2 and 7.5 Å, respectively. 

To ensure that PEG moieties that are present in the outer lipid leaflet in the *t*-BLM were not responsible for the stronger AmpA association onto the *t*-BLM in comparison with the HBM, AmpA binding properties were also evaluated using an HBM formed by the fusion of vesicles containing eggPC and DSPE-PEG NHS (5% w). The association levels and kinetic curves were unchanged compared to a pure eggPC HBM (data not shown). 

#### 3.2.2. Lipid Fluidity Modulation by AmpA

**Figure 5:**
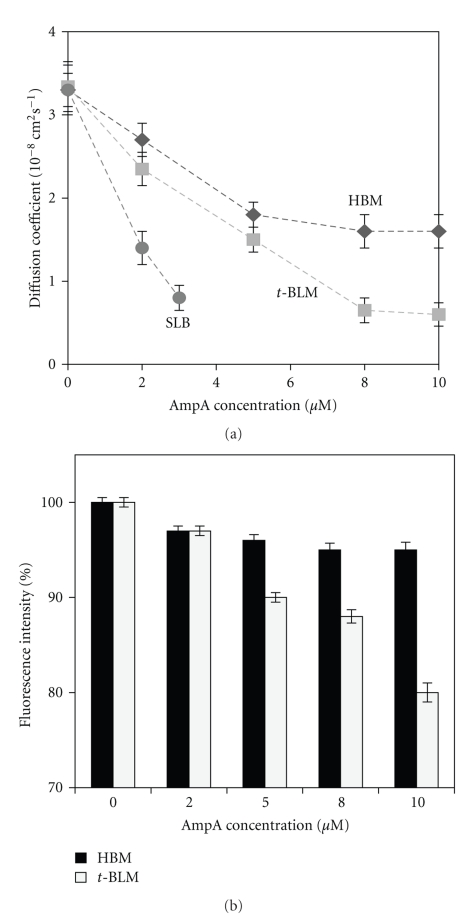
Lipid mobility changes associated with AmpA interaction with membranes. (a) Overlay plots of the modification of the lipid diffusion coefficient as a function of AmpA concentration in the SLB, the HBM, and the *t*-BLM. Measurements were performed within an AmpA concentration range from 0 to 10 *μ*M with the HBM, and the *t*-BLM and from 0 to 3 *μ*M with the SLB. (b) Fluorescence emission intensity of NBD-DPPE incorporated in the lipid layers as a function of AmpA concentration at *t* = 2*s* after peptide injection. The fluorescence intensity was normalized at 100% for all the lipid layers before peptide injections.

AmpA effect on lipid dynamics was quantified by FRAP analysis. Three biomimetic membrane models were used in this study: the HBM, the *t*-BLM, and the SLB that corresponds to a free-standing bilayer. The comparison of AmpA association effects on lipid diffusion in the three models was expected to allow testing the putative influence of the thickness of the aqueous reservoir beneath the lipid layers (reduced in the SLB) and of the covalent linkages between the inner bilayer leaflet and the support present in the HBM and the *t*-BLM but not in the SLB. The three structures possess an identical initial lipid diffusion coefficient *D* of 3.5 ± 0.4 ×10^−8^ cm^2^·s^−1^, in agreement with the previously published data [47, 48]. AmpA significantly modulated lipid fluidity (Figure 5(a)). The injection of AmpA at concentrations below 5 *μ*M triggered a ~2-3-fold reduction of the lipid diffusion coefficient in both the HBM and the *t*-BLM. Above 5 *μ*M AmpA, the diffusion coefficient in the *t*-BLM *D*_*t*-BLM_ dropped to 0.6 ± 0.1 ×10^−8^ cm^2^·s^−1^ (i.e., overall reduction by 83% compared to the initial value) while the diffusion coefficient in the HBM *D*_HBM_ remained constant at *D*_HBM_ = 1.6 ± 0.2 × 10^−5^  cm·s^−1^ (overall decrease by 54%). The SLB fluidity was the most strongly affected showing a decrease by 75% compared to its initial value for a concentration of 3 *μ*M of AmpA. 

Figure 5(b) summarizes the fluorescence intensity of the nonbleached area surrounding the photobleached spot at *t* = 2 *s* after AmpA injection at different concentrations (Figure 5(b)). Only a little loss of fluorescence was observed in the HBM, while the fluorescence intensity significantly decreased for the *t-*BLM structure. Indeed in the latter one, the ratio *F*/*F*_0_ (fluorescence intensity/peptide-free fluorescence intensity) was 0.9 and 0.8 for AmpA concentrations of 5 *μ*M and 10 *μ*M, respectively. These data were in line with a substantial departure of fluorescent probes from the lipid bilayers after their interaction with AmpA.

## 4. Discussion

AmpA and other medium-sized peptaibols (14-15 amino acid residues) share common and specific activities such as the induction of pigment formation by the fungus *P. destructiva* and long acting hypothermia and depression of locomotor activity in mice [17, 31]. These biological effects have previously been correlated with membrane voltage-dependent pore formation [31]. Nevertheless, AmpA pore formation activity observed upon voltage appliance in soybean phosphatidyl choline planar bilayers is several orders of magnitude lower compared to Alm [31]. Thus, the voltage-dependent pore formation capacities of AmpA may not be sufficient to explain its specific bioactivities, and an additional, voltage-independent membrane mechanism may occur. The aim of this study was to better understand AmpA interaction properties with biological membranes and to elucidate whether AmpA was able to efficiently act independently of a transmembrane voltage appliance or not. To fulfill this goal, AmpA association was investigated using different lipid environments to evaluate its membrane-disrupting properties in the absence of membrane voltage. 

### 4.1. Binding to and Insertion in the Outer Lipid Leaflet

Using BAM associated with surface pressure-area isotherms, it was shown that the thickness of the layer formed at the air/HEPES-NaCl phase interface by the peptide alone (in the absence of lipids) was in agreement with a helical conformation of AmpA lying flat on the surface. At high AmpA concentrations, the occurring spots were likely due to the self-aggregation of peptide helices at the interface. A series of experiments carried out with Langmuir monolayers at the air/water interface were proved to be particularly informative for understanding the first steps of the mechanism of AmpA-membrane interaction. Examples of similar investigations on Langmuir monolayers for studying peptide-membrane interactions have been former studies performed with the fusion peptide FP23 [59] and of antimicrobial representative peptides [60, 61]. When AmpA was injected in the subphase underneath the eggPC Langmuir monolayer, the molecular area rose in agreement with AmpA binding to- and insertion into the PL monolayer. For 20 nM AmpA, the area increase of 4.3 A^2^/molecule indicated that almost 10% of the initial lipid surface was occupied by the peptide (assuming that the lipid molecular area does not significantly vary during AmpA interaction), which means that AmpA deeply penetrated the lipid layer. The slight increase of the GL in BAM images, which is directly related to the optical thickness of the mixed AmpA-lipid monolayer, was found to be coherent with a small amount of AmpA adsorbed on the lipid surface. Thus, AmpA/membrane interaction at low AmpA concentration resulted from a two-step mechanism: (1) binding and (2) insertion of AmpA molecules into the eggPC monolayer core. PMIRRAS spectra indicated that AmpA interacted with the monolayer and modified the IR spectra of the PL polar head group. This event could result from the association of AmpA with the PL polar moieties; this could be followed by the PL reorganization and/or from hydrophobic interactions between AmpA and the lipid layer's hydrophobic core, as it was previously shown to occur for Alm in liposomes [62]. PMIRRAS studies also provided clues about AmpA orientation and conformation in the membrane outer leaflet. The high intensity of the amide I band at about 1665 cm^−1^ indicated that AmpA was present at the interface with a predominant *α*-helical conformation. Nevertheless, the high frequency (1665 cm^−1^) and the shape (shoulder, 1670 cm^−1^) of the amide I band suggested a partial 3_10_-helical conformation of the peptide when mixed with the lipids. While the peptide 3_10_-helix conformation is relatively uncommon in proteins [55], it is very common in peptaibols. The high intensity ratio of the amide I/amide II bands due to the reduced amide II band around 1538 cm^−1^ was coherent with a mainly flat-oriented helix at the interface. These data are in agreement with former studies indicating that AmpA preferentially adopts an helical conformation in crystal [33] and in membrane mimicing environments [36, 63]. AmpA has also a mixed helical structure in a transmembrane (TM) orientation in thin artificial bilayers and under these conditions, the partial 3_10_ conformation has been proposed to be due to the restraints imposed to the peptaibol by the bilayer hydrophobic core [36]. In monolayers, AmpA kept a nearly *in plane* flat orientation, indicating that a mixed *α*/3_10_-helical structure can also be associated with a non-TM conformation. At high AmpA concentrations injected in the subphase, the monolayer disruption was correlated to a decrease of the GL in BAM images of the interface. These data are coherent with the departure of peptide/lipids mixed aggregates from the monolayer following the monolayer disruption.

### 4.2. Importance of the Inner Lipid Leaflet on AmpA Membrane Binding Properties

To elucidate the subsequent steps of AmpA membrane interaction without voltage appliance, FRAP and SPR analyses were carried out on HBM, *t*-BLM, and SLB. SPR spectroscopy has previously proved to be useful for interaction studies of cytolytic peptides with membranes [40, 64–68]. More particularly, real-time measurements using the Biacore system were used to discriminate between the “detergent-like” mechanism and the formation of more defined pores by membranotropic peptides, on the basis of the comparison between the affinity constants using HBMs and supported lipid bilayers (immobilized liposomes or planar bilayers) [40, 65]. In the present study, advantage was taken of the structural differences between the HBM and the *t*-BLM. An essential difference between the two models consists in the *trans* leaflet which is a rigid alkane layer in the HBM and a fluid lipid layer in the *t*-BLM. In contrast to the HBM, the lipid bilayer provides a hydrophobic core, which can be compared to the hydrophobic core of biological membranes. In addition, the *t*-BLM model relies on the covalent attachment of the bilayer to the gold support *via* PEG spacer molecules (77 ethylene glycol units) and allows the creation of a *trans* compartment underneath the bilayer [47, 48]. These two planar membrane models offer the possibility to perform SPR and FRAP measurements under similar experimental conditions [47]. Furthermore, both models exhibit an identical flat lipid surface that allows the direct comparison of SPR and FRAP signals in order to answer the question of the role of the *trans* lipid leaflet in the AmpA/membrane association process. Given that AmpA binding behaviour was identical in HBM constructs that contain or not DSPE-PEG-NHS, it was assumed that the PEG chains had no influence on AmpA binding in this experimental setup.

Unfortunately, although AmpA kinetics were highly reproducible, fitting the reflectivity curves to a simple 1 : 1 binding model or to a two-step binding thickness model did not give satisfying results and no association/dissociation constant could be extracted from the SPR data. The difficulty to fit mathematical models to the experimental data suggested that the hydrophobic AmpA acts on membranes following a distinct mechanism from that of the amphipathic, cationic peptides for which relevant binding affinity constants have been successfully determined by SPR [40]. Therefore, the peptide interaction with supported membranes was investigated in the present work by comparing the structural changes in fluidity and optical thickness occurring upon AmpA membrane interaction in the different membrane systems.

AmpA binds both supported membranes in the absence of voltage. AmpA binding led to a drastic decrease of the lipid lateral diffusion coefficient, reflecting the restraints imposed by AmpA-PL interactions. As illustrated by the association curves of AmpA ([AmpA] ≤ 5 *μ*M) to the HBM and to the *t-*BLM using SPR (Figure 4), the amount of bound peptides was more important in the *t*-BLM, that is, in the presence of two lipid leaflets. This result was confirmed using FRAP, as the effect of AmpA addition on the lateral diffusion of the DPPE-NBD molecules was more pronounced on the *t-*BLM. Two main hypotheses could be brought to explain the effect of the presence of an inner lipid leaflet on the association process in agreement with an initial *in-plane* orientation of AmpA in the outer lipids: (1) AmpA monomers interact with the hydrophobic core of the *trans* lipid leaflet and the induced structural changes at the surface favour the binding of additional peptide monomers, and/or (2) the peptide reaches the *trans* aqueous reservoir *via* radial diffusion and interacts with the *trans* PL leaflet. 

The SLB model provided an interesting tool for testing the second hypothesis. In this lipid bilayer model, the water layer thickness between the surface and the bilayer is reduced to 1-2 nm [69, 70]. This configuration prevents the peptide diffusion from the aqueous buffered phase and its direct interaction with the *trans* surface, as assumed in hypothesis 2. The intermolecular forces between the bilayer and the support are of low energy for the SLB, while the *t*-BLM possesses covalently anchored lipids. In the HBM, all the molecules forming the inner leaflet are covalently linked to the support. AmpA effect on lipid fluidity was more important in the SLB in comparison with the *t*-BLM. Thus, the high binding level obtained with the *t*-BLM could hardly be attributed to a direct access of the peptide to the *trans* surface *via* radial aqueous diffusion. Moreover, the binding step was highly influenced by the inner leaflet rigidity, which is related to the strength of the interaction between the inner leaflet and the support. Taken together, these data are in line with hypothesis 1 stated above and indicate that the inner leaflet may participate in AmpA binding and insertion through AmpA-lipid interactions.

### 4.3. Membrane Disruption

Upon AmpA interaction with the lipids at the air/water interface, the PL layer collapse pressure decreased by about 5 mN·m^−1^ when 20 nM AmpA were injected. This event may be attributed to the presence of aggregates in the monolayer and its subsequent weakening. This assumption is consistent with the lipid departure from the surface observed once a critical injected peptide concentration (likely corresponding to a critical peptide concentration in the monolayer) was reached. AmpA also induced the lipid leakage from lipid bilayers. Above this critical peptaibol concentration, AmpA-membrane interaction was associated with a drastic decrease of total fluorescence intensity of NBD-labelled lipid containing bilayers. This loss of fluorescence was very probably due to the transfer of NBD-labelled lipids from the bilayer into the aqueous environment. All these data are in agreement with an AmpA induced membrane solubilization process that occurred in the *t-*BLM and not in the HBM. Finally, considering our SPR and FRAP measurements, the inner lipid leaflet contributed to the structural changes accompanying the binding/insertion process, but also to the bilayer solubilization event. 

## 5. Conclusions

In the absence of voltage, AmpA was found to be flat-oriented in eggPC monolayers at the air/water interface. In eggPC vesicles, a small fraction of bound AmpA is also involved in the formation of a transmembrane state [32]. Thus, the supported membrane disorganization triggered by the peptaibol unlikely results from a TM orientation of AmpA monomers in the membrane and is thus inconsistent with the formation of well-defined pores. The model combining a surface “carpet-like” association of peptide monomers and toroidal pore formation [2, 3] is commonly used to explain the mechanism of membranolytic peptides bearing an amphipathic and cationic character (e.g. melittin, magainin). Various “detergent-like” effects of antimicrobial peptides on membranes can be distinguished [71], and the combined “carpet-toroidal” model has to be slightly modified to fit the present experimental data obtained with AmpA. Upon a carpet-like peptide association, the peptide monomers adsorb on the surface *via* electrostatic interactions between the cationic amino acids and the negatively charged PL heads. AmpA mechanism towards eggPC bilayers includes its adsorption at the surface but also its insertion into the hydrophobic core of the outer lipid leaflet with an initial nearly in membrane-plane orientation. The high AmpA hydrophobicity and the lack of any charged residue in its sequence are very probably responsible for its ability to insert into the hydrophobic core of the membrane. The monomers then incorporate into the membrane and very probably interact with the inner PL leaflet, creating weakened areas in the membrane. Above a critical AmpA concentration, the formation of AmpA/lipids mixed aggregates leads to membrane disruption. In conclusion, AmpA *in vivo* likely acts through both a voltage-dependent pore formation and a non-TM insertion that does not require a transmembrane voltage and that may lead to membrane disruption. Nevertheless, the participation of its voltage-dependent pore formation abilities [31], its voltage-independent membrane organization-disrupting effects (the present study), or even its eventual interaction with receptors that has recently been proposed [63] in the different bioactivities will have to be considered in future investigations. Finally, the complementarity as well as the specific features of the supported membrane models HBM, SLB and *t*-BLM proved to offer great interests when attempting to decipher the mechanism of membranotropic peptides with lipid membranes, even when membrane solubilization occurs. 

## Figures and Tables

**Table 1:** Optical thickness measured by SPR after the interaction of AmpA at increasing concentrations with the biomimetic lipid membranes.

	Peptide layer thickness (Å)^(a)^											
	0.5 *μ*M		1 *μ*M		2 *μ*M		5 *μ*M		8 *μ*M		10 *μ*M	
	Before	After	Before	After	Before	After	Before	After	Before	After	Before	After

HBM	0	0	0.5 ± 0.5	0	0.5	0	2 ± 0.5	0	2.5 ± 0.5	0	2.5 ± 0.5	0
*t*-BLM	0	0	1.5 ± 0.5	0	3 ± 0.4	0	5 ± 0.4	1 ± 0.5	—	Membrane disruption	—	Membrane disruption

^(a)^The baseline was attributed to the lipid membrane thickness before peptide injections. The thickness values are given before and after the buffer rinsing step annotated *R* in Figure 4.
